# The Common Effects of Sleep Deprivation on Human Long-Term Memory and Cognitive Control Processes

**DOI:** 10.3389/fnins.2022.883848

**Published:** 2022-06-02

**Authors:** Taehyun Kim, Sejin Kim, Joonyoung Kang, Minjae Kwon, Sue-Hyun Lee

**Affiliations:** ^1^Department of Bio and Brain Engineering, College of Engineering, Korea Advanced Institute of Science and Technology, Daejeon, South Korea; ^2^Program of Brain and Cognitive Engineering, College of Engineering, Korea Advanced Institute of Science and Technology, Daejeon, South Korea

**Keywords:** sleep deprivation, circadian rhythm, long-term memory, cognitive control, light

## Abstract

Sleep deprivation is known to have adverse effects on various cognitive abilities. In particular, a lack of sleep has been reported to disrupt memory consolidation and cognitive control functions. Here, focusing on long-term memory and cognitive control processes, we review the consistency and reliability of the results of previous studies of sleep deprivation effects on behavioral performance with variations in the types of stimuli and tasks. Moreover, we examine neural response changes related to these behavioral changes induced by sleep deprivation based on human fMRI studies to determine the brain regions in which neural responses increase or decrease as a consequence of sleep deprivation. Additionally, we discuss about the possibility that light as an environmentally influential factor affects our sleep cycles and related cognitive processes.

## Introduction

As indoor lifestyles dominate and the variability of active hours increases, our daily sleep schedule becomes less dependent on environmental cues such as sunlight. For example, a substantial number of people work day and night shifts, such as a nurse’s night shift. In such cases, sleep deprivation can be induced due to a disturbance in the circadian rhythm (Dijk et al., 1997; Hou et al., 2019). In addition, jet lag from air travel or internal emotional states such as excessive stress can also cause sleep deprivation (Weingarten and Collop, 2013). Here, sleep deprivation indicates getting less than the required amount of sleep, which typically between 7 and 9 h of sleep per night for adults (Hirshkowitz et al., 2015). Sleep deprivation can occur for short periods of time, such as 1 day (acute sleep deprivation), or for long periods, such as a few months or longer (chronic sleep deprivation) (Sateia, 2014). Sleep deprivation not only involves physiological changes throughout the entire body but is also considered to be critically related to the decline of a range of cognitive functions (Durmer et al., 2005; Ratcliff and van Dongen, 2009; Jackson et al., 2013; Lo et al., 2016). Moreover, causal relationships between sleep loss and mental disorders, including ADHD, depression, bipolar disorder, and schizophrenia, have been reported (Harvey et al., 2005; Murray and Harvey, 2010; Baird et al., 2012; Wulff et al., 2012; Roberts and Duong, 2014; Yu et al., 2016).

Over the past few decades, there has been a great deal of research on the effects of sleep deprivation on various cognitive functions (Krause et al., 2017). One of the most commonly reported cognitive functions affected by sleep is the memory function (Abel et al., 2013). In particular, it has been suggested that there are critical processes for memory consolidation during slow wave sleep (SWS, Stage 3 and 4) (Plihal and Born, 1997; Rasch et al., 2007; Walker, 2009; Born, 2010; Antony et al., 2012; Cox et al., 2012) or the rapid eye movement (REM) (Plihal and Born, 1997; Wagner et al., 2001; Hornung et al., 2007; Rasch and Born, 2013; Boyce et al., 2016) sleep stage, leading to long-term memory (LTM) formation. Although the distinctive role of each sleep stage is still controversial, cortical activity during those stages of sleep is proposed to be associated with the replay or reinstatement process of experiences to strengthen memories (Pennartz et al., 2002; Paller and Voss, 2004; Antony et al., 2012; Schreiner and Rasch, 2017). Moreover, sleep deprivation has been found to be related to the impairment of various types of LTM formation and to decreases of retrieval performance (Stickgold et al., 2000a; Walker et al., 2002; Korman et al., 2003; Drosopoulos et al., 2005; Gais et al., 2006; van der Helm et al., 2011; Griessenberger et al., 2012). The cognitive control process is also known to be influenced by sleep deprivation (Krause et al., 2017). The overall arousal level is decreased after sleep deprivation, and the corresponding changes usually cause declines in cognitive control functions such as attention and working memory processes (Chee and Choo, 2004; Versace et al., 2006; Kuriyama et al., 2008; Chee et al., 2010).

In this review, we focus on prior human research that addresses the influence of sleep deprivation on long-term memory and cognitive control processes. We used Google Scholar and PubMed to search the literature containing the main keywords “sleep deprivation,” “human,” and “long-term memory (or working memory or executive/cognitive control)” since 2000, and selected papers that report changes in human behaviors and neural responses before and after sleep deprivation. Most of these studies have focused on the effects of acute sleep deprivation rather than chronic sleep deprivation.

In the first and second sections, we summarize behavioral changes and neuronal changes induced by sleep deprivation, which are commonly reported across prior studies despite the use of various types of stimuli and task paradigms. In addition, we discuss influential factors affecting sleep and related cognitive functions.

## Long-Term Memory and Sleep Deprivation

Long-term memory (LTM) paradigms usually involve more than 1 day of sleep between learning and retrieval. The beneficial effects of sleep on LTM were found in the early 20th century, and those studies have been replicated numerous times (Drosopoulos et al., 2005; Gais et al., 2006; Aly and Moscovitch, 2010; van der Helm et al., 2011). Specifically, memory researchers have focused on the role of sleep in the memory consolidation process (Abel et al., 2013). Prior studies suggest that neural activity during slow wave sleep (SWS, Stage 3 and 4) or REM sleep stage is associated with the replay of past experiences, contributing to the consolidation process of memory traces (Pennartz et al., 2002; Paller and Voss, 2004; Antony et al., 2012). Thus, sleep deprivation may adversely affect memory consolidation. Here, we examine the effect of sleep deprivation on declarative memories, in which the hippocampus plays a key role, and on non-declarative memories, where other areas rather than the hippocampus are thought to be critical.

### Declarative Memories

The formation and consolidation of declarative memories, including verbal, visual, episodic memories, critically involve hippocampal processes (Tulving and Markowitsch, 1998; Eichenbaum, 1999, 2004; Manns and Eichenbaum, 2006). Sleep studies have shown hippocampal reactivation in the form of sharp wave ripples during sleep, which is thought to be critically associated with memory consolidation (Ji and Wilson, 2007; Eschenko et al., 2008; Ramadan et al., 2009; Born, 2010; Diekelmann and Diekelmann and Born, 2010). Therefore, it is expected that sleep deprivation will negatively affect hippocampus-dependent consolidation processes for declarative memories during sleep.

Numerous studies of the effects of sleep deprivation on declarative memory used verbal memory paradigms (Drosopoulos et al., 2005; Gais et al., 2006; Ellenbogen et al., 2009; Feld et al., 2016) (Table 1). Verbal stimuli such as words or nonsense syllables are useful for controlling the variability between stimuli and for generating false memories based on semantic contents. Sleep deprivation has been found to reduce memory performance in simple word recognition tasks (Drosopoulos et al., 2005). Other studies used word pairs to test memory function and showed similar decreases in sleep deprived groups (Gais et al., 2006; Ellenbogen et al., 2009; Feld et al., 2016). Some sleep deprivation studies used a semantically related word list, as in the Deese-Roediger-McDermott (DRM) paradigm to generate false memories. They found that sleep deprivation not only reduces correct trials but also increases false alarm trials (Diekelmann et al., 2008; Fenn et al., 2009). Additionally, Ellenbogen et al. found that sleep deprivation makes memory vulnerable to post-learning interference (Ellenbogen et al., 2009).

**TABLE 1:** Studies of the effects of sleep deprivation on declarative memories.

	References	Stimuli	Number of stimuli	*N*	Comparison/Sleep-deprivation condition	Results
Verbal memory	Drosopoulos et al. (2005)	Word	96 words	24	Sleep vs. Wake /3 h deprivation	Accuracy: sleep > wake, early > late
	Ellenbogen et al. (2009)	Word pair	60 pairs	45	Sleep vs. Wake /12 h deprivation	Accuracy: sleep > wake
	Feld et al. (2016)	Word pair	160/320 pairs	101	Sleep vs. Wake /8 h deprivation	Accuracy: sleep > wake (160 pairs)
	Gais et al. (2006)	Word pair	24 pairs	14	Sleep vs. Wake /12 h deprivation	Accuracy: sleep > wake
	Diekelmann et al. (2008)	Word (DRM)	270 words	43	Sleep vs. Wake /9 or 33 h deprivation	False memory: sleep < wake (9 h), sleep < wake (33 h)
	Fenn et al. (2009)	Word (DRM)	240 words	46	Sleep vs. Wake /12 h deprivation	False memory: sleep < wake
	Payne et al. (2009)	Word (DRM)	120 words	84	Sleep vs. Wake /12 h deprivation	Accuracy: sleep > wake
	Lutz et al. (2017)	Abstract shape (DRM)	160 shapes	16	Sleep vs. Wake /10 h deprivation	Confidence: sleep > wake
Non-verbal memory	Prehn-Kristensen et al. (2009)	IAPS image	120 images	20	Sleep vs. Wake /11 h deprivation	Accuracy: sleep > wake
	Rauchs et al. (2004)	Sequence and Location of words	14 words	61	Sleep vs. Wake /4 h deprivation	Accuracy: sleep > REM deprivation
	Drosopoulos et al. (2007)	Sequence of words	32 words	28	Sleep vs. Wake /9 h deprivation	Accuracy: sleep > wake
	Aly and Moscovitch (2010)	Short-stories	2 stories	22	Sleep vs. Wake /12 h deprivation	Accuracy: sleep > wake
	Griessenberger et al. (2012)	Picture sequence	8 sequences	34	Sleep vs. Wake /24 h deprivation	Accuracy: sleep > wake

*DRM, Deese-Roediger-McDermott paradigm; IAPS, International Affective Picture System; h, hours.*

Other studies based on visual memory paradigms, in which participants memorize abstract shapes or pictures, also revealed lower performance in a sleep-derivation group compared to a group with a normal amount of sleep (Prehn-Kristensen et al., 2009; Lutz et al., 2017). Episodic memory studies have also shown negative effects of sleep deprivation on memory performance (van der Helm et al., 2011; Inostroza and Born, 2013; Tempesta et al., 2017; Chai et al., 2020). Sleep deprivation disrupts the formation of temporal information for both verbal and visual stimuli (Rauchs et al., 2004; Drosopoulos et al., 2007; Griessenberger et al., 2012). Moreover, van der Helm et al. showed that contextual memory was specifically impaired in a sleep-deprivation group even when verbal item memory remained intact (van der Helm et al., 2011).

While most studies of the effect of sleep deprivation on memory address the effects of overnight-sleep deprivation, there is also evidence supporting that 3–4 h-sleep restriction can also affect memory performance (Rauchs et al., 2004; Drosopoulos et al., 2005). Thus, even partial sleep deprivation can affect memory formation and subsequent retrieval.

### Non-declarative Memories

Non-declarative memory, often referred to as implicit memory, is an unconscious form of memory that is typically manifested in an automatic manner (Squire and Zola, 1996). Procedural memory, including motor memory and perceptual skills, is one of the most common forms of non-declarative memory. Such memories are known to be associated with neural substrates distinct from those of declarative memories and are thus usually residual, even in patients with amnesic disease or hippocampal lesions (Cohen and Squire, 1980; Döhring et al., 2017; Corkin, 2022). It is known that regions other than the hippocampus, such as cerebellum and striatum, are mainly involved in non-declarative memory processes (Squire and Zola, 1996; Doyon et al., 1997, 2003).

Motor memory refers to memory involving motor skills, such as playing an instrument. In many motor memory tasks, memory performance is evaluated based on sequential finger tapping movements. Studies of sleep deprivation using this finger tapping paradigm have found that performance decreases in a sleep-deprived condition (Walker et al., 2002; Korman et al., 2003; Debas et al., 2010) (Table 2). In addition, Korman et al. showed that sleep enhances resistance to post-learning interference tasks such as inverse-tapping-sequence learning. Another study also found a motor memory decline after sleep deprivation based on the mirror task paradigm, in which participants were asked to trace drawings by looking in a mirror to observe a picture to be traced.

**TABLE 2:** Studies of the effects of sleep deprivation on non-declarative memories.

	References	Task type	Stimuli	*N*	Comparison/Sleep-deprivation condition	Results
Motor memory	Walker et al. (2002)	Finger tapping task	5 element sequence	62	Sleep vs. Wake /12 h deprivation	Accuracy: sleep > wake
	Korman et al. (2003)	Finger tapping task	5 element sequence	83	Sleep vs. Wake /12 h deprivation	Accuracy: sleep > wake
	Debas et al. (2010)	Finger tapping task	5 element sequence	48	Sleep vs. Wake /12 h deprivation	Accuracy: sleep > wake
Perceptual memory	Aeschbach et al. (2008)	Visual texture discrimination task	T and L	16	Sleep vs. Disruption /4 h deprivation	Accuracy: sleep > disruption
	Stickgold et al. (2000b)	Visual texture discrimination task	T and L	57	Sleep vs. Wake /8 or 12 h deprivation	Accuracy: sleep > wake
	Stickgold et al. (2000a)	Visual texture discrimination task	T and L	11	Sleep vs. Wake /24 h deprivation	Accuracy: sleep > wake
	Mascetti et al. (2013)	Visual orientation discrimination task	Grating	32	Sleep vs. Wake /12 h deprivation	Accuracy: sleep > wake

*h, hours.*

Prior studies based on perceptual skill memory paradigms, mostly visual discrimination tasks, have also shown reduced performance outcomes under a sleep-deprived condition (Stickgold et al., 2000a,b; Aeschbach et al., 2008; Mascetti et al., 2013). Notably, perceptual skill memory has been shown to decline simply after disrupting sleep quality with the same amount of total sleep time (Aeschbach et al., 2008) (Table 2). Moreover, as with declarative memory, relatively short durations of sleep deprivation also affect the procedural memory performance outcomes (Aeschbach et al., 2008).

These results indicate that not only declarative memories but also various types of non-declarative memories are negatively affected by sleep deprivation, despite the fact that these memories are known to depend less on hippocampal consolidation (Döhring et al., 2017; Corkin, 2022). In sum, sleep deprivation adversely affects our overall memory performance, including both declarative and non-declarative memory processes.

## Cognitive Control and Sleep Deprivation

Cognitive control (or executive control) is also known to be especially susceptible to sleep deprivation (Krause et al., 2017). Previous studies have found that sleep deprivation also negatively affects cognitive control processes (Table 3). Sleep-related changes in cognitive control are important because such changes can affect our overall goal-dependent behaviors (Paxton et al., 2008; Braver, 2012; Nathan Spreng et al., 2014). In this section, we focus on the effects of sleep deprivation on working memory and attention processes.

**TABLE 3:** Studies of the effects of sleep deprivation on cognitive control processes.

	References	Task type	Stimuli	*N*	Comparison/ Sleep-deprivation condition	Results
Working memory	Chee and Choo (2004)	Delayed match-to-sample	Alphabet letter	14	Sleep vs. Wake /24 h deprivation	Accuracy: sleep > wake
	Mu et al. (2005a)	Delayed match-to-sample	Alphabet letter	33	Sleep vs. Wake /30 h deprivation	Reaction time: sleep < wake Accuracy: sleep > wake
	Luber et al. (2008)	Delayed match-to-sample	Alphabet letter	15	Sleep vs. Wake /57 h deprivation	Reaction time: sleep < wake
	Drummond et al. (2012)	Delayed match-to-sample	Color	44	Sleep vs. Partial sleep vs. Wake /4 or 24 h deprivation	No difference
	Xie et al. (2019)	Delayed match-to-sample	Color	110	Sleep quality	Positive correlation between sleep quality and WM performance
	Kuriyama et al. (2008)	N-back task	Button image	29	Sleep vs. Wake /10 h deprivation	WM capacity: sleep > wake
	Gradisar et al. (2008)	Delayed recall	Word	143	Sleep vs. Restricted sleep /8 h sleep restriction	Accuracy: restricted sleep < sleep
Attention	Mander et al. (2008)	Spatial attention task	Orientation	7	Sleep vs. Wake /36 h deprivation	Commission error: no difference Omission error: sleep < wake
	Versace et al. (2006)	Spatial attention task	Orientation	14	Sleep vs. Restricted sleep /5 h sleep restriction	Reaction time: sleep < restricted sleep (invalid cue)
	Bocca and Denise (2006)	Saccade task	Dots	10	Sleep vs. Wake /24 h deprivation	Latency: sleep < wake
	Chee et al. (2010)	Object-selective attention task	Scene, face stimuli	26	Sleep vs. Wake /24 h deprivation	Reaction time: sleep < wake, Accuracy: sleep > wake,
	Jennings et al. (2003)	Go/No-Go task	Orientation	20	Sleep vs. Wake /12 h deprivation	Reaction time: sleep < wake Accuracy: sleep > wake (incompatible cue)
	Nota and Coles (2018)	Attention disengagement task	Emotional images	52	Sleep duration	Shorter sleep duration correlated with slower disengaging from negative stimuli
	Fallone et al. (2001)	The child attention profile	Questionnaire	82	Sleep vs. Restricted sleep /6 h sleep restriction	Inattentive behavior: restricted sleep > sleep

*WM, working memory; h, hours.*

### Working Memory

Working memory is a cognitive ability that allows one to hold information temporarily to guide current behavior (Baddeley and Hitch, 1974; Lee and Baker, 2016). The delayed match-to-sample (DMTS) task is the most widely used paradigm to test working memory. In prior verbal working memory studies based on DMTS, sleep deprivation generally decreases accuracy levels and increases reaction times (Chee and Choo, 2004; Mu et al., 2005a; Luber et al., 2008). However, in visual working memory studies, discrepancies were reported with regard to the effects of sleep deprivation. While some studies show a performance decline after sleep deprivation (Xie et al., 2019), others show no differences between sleep and waking conditions (Drummond et al., 2012). In addition, MacDonald et al. reported a temporal difference in the effect of sleep deprivation; no group difference was found in the early phase of the working memory task, while a performance decline in a sleep-deprived group was found in the late phase of the task (MacDonald et al., 2018). Because working memory may be mainly affected by increased fatigue or the altered brain states induced by sleep deprivation, momentary focusing may compensate for the decline of performance from sleep deprivation. Consistent with this idea, some studies reported no differences between groups assessed on difficult or complex task conditions which involve intentional effort (Chee and Choo, 2004; Mu et al., 2005a). Thus, it may be possible that the effect of sleep deprivation on working memory depends on the balance or interaction between the attention process and sleep-deprivation-induced changes.

### Attention

Sleep-deprivation effects have been reported in various attention studies. Bocca and Denise used a simple saccade task to evaluate sustained attention and found longer latency times in a sleep-deprived group (Bocca and Denise, 2006). On a selective-attention task requiring high top-down regulation, performance declines in sleep-deprived groups with an orientation cue (Jennings et al., 2003; Versace et al., 2006; Mander et al., 2008), and an object cue (Chee et al., 2010). Furthermore, performance changes during attention processing were found to occur even with differences in short and long sleep durations (Fallone et al., 2001; Versace et al., 2006). Additionally, there are numerous reports of sleep deprivation effects, especially when inhibitory top-down control is required (Jennings et al., 2003; Versace et al., 2006; Mander et al., 2008; Nota and Coles, 2018).

Taken together, these studies mostly suggest sleep-deprivation-induced declines in the performance outcomes of cognitive control processes. However, compared to the effects on long-term memory, the effects of sleep deprivation on the cognitive control process depend more sensitively on the experimental conditions.

## Neural Response Changes Induced by Sleep Deprivation

The aforementioned studies suggest that sleep deprivation induces declines in memory and executive control performances in common, despite the different task paradigms utilized. This leads to the question of what neural bases underlie the cognitive decline induced by sleep deprivation. In this section, we focused on human fMRI studies that investigated the effects of sleep deprivation on memory and executive control processes. Sleep-derivation associated activity changes were commonly observed in the prefrontal cortex, parietal lobe, hippocampus, and basal ganglia/thalamus.

### Prefrontal Cortex

Changes of neural responses in the prefrontal cortex are reliably observed in the human fMRI literatures (Table 4). In the lateral prefrontal areas, including the dorsolateral prefrontal cortex (dlPFC) and ventrolateral prefrontal cortex (vlPFC), decreased neural responses as a result of sleep deprivation have often been reported (Mu et al., 2005b; Lythe et al., 2012; Wang et al., 2016). In some studies, sleep-loss-associated increases in activity in the dlPFC were also reported (Chee and Choo, 2004; Beebe et al., 2009; Drummond et al., 2012). Consistent with the fact that the PFC regions are mainly involved in working memory, attention and executive control processes (Wagner et al., 2001; Curtis and D’Esposito, 2003; Barbey et al., 2013), the response changes in the PFC as a consequence of sleep deprivation were mostly observed in the studies using working memory tasks (Mu et al., 2005b; Chee et al., 2006; Lythe et al., 2012) (Table 4).

**TABLE 4:** Human fMRI studies of the effects of sleep deprivation on neural response changes.

References	Sleep deprivation	Cognitive process	*N*	Neural response changes	
				Decrease	Increase
Drummond et al. (2000)	35 h deprivation	Short-term verbal memory task	13	Temporal lobe	PFC, parietal lobe
Habeck et al. (2004)	48 h deprivation	Working memory task	18	Parietal, temporal, occipital lobe	Cerebellum, basal ganglia, thalamus, Anterior cingulate cortex (ACC)
Bell-McGinty et al. (2004)	48 h deprivation	Short-term non-verbal memory task	19	Bilateral posterior cerebellum, right fusiform gyrus, precuneus, left lingual and inferior temporal gyri	Bilateral insula, claustrum, tight putamen
Chee and Choo (2004)	24 h deprivation	Working memory task	14	Anterior medial frontal, left posterior cingulate, medial parietal region	Dorsal lateral prefrontal cortex (dlPFC)
Mu et al. (2005b)	30 h deprivation	Working memory task	20	Left posterior parietal cortex (PPC), left ventro lateral prefrontal cortex (vlPFC), right inferior PPC, right dlPFC	
Chee et al. (2006)	24 h, 35 h deprivation	Working memory task	26	Left lPFC, ACC, bilateral superior parietal region, left thalamus	
Lim et al. (2007)	24 h deprivation	Working memory task	19	Bilateral parietal region	
Gais et al. (2007)	24 h deprivation	Long-term verbal memory task	18	Hippocampus (HPC)	
Yoo et al. (2007)	35 h deprivation	Long-term episodic memory task	28	Bilateral posterior HPC	
Chee and Chuah (2007)	24 h deprivation	Working memory task	30	Precuneus, temporoparietal junction (TPJ)	
Beebe et al. (2009)	Chronic deprivation	Working memory task	6	Left postcentral gyrus, lingual gyrus, ACC, middle frontal gyrus (MFG), PCC, parahippocampal gyrus, fusiform gyrus, superior temporal gyrus, middle temporal gyrus	Precuneus, premotor, dlPFC, insula, inferior parietal lobe (IPL), cingulate gyrus
Van Der Werf et al. (2009)	Disturbed sleep	Long-term visual memory task	13	Anterior HPC	
Gujar et al. (2010)	36 h deprivation	Attention task	28	Precuneus	Dorsal ACC
Lim et al. (2010)	24 h deprivation	Attention task	23	Left inferior frontal cortex, left intraparietal sulcus (IPS)	
Jackson et al. (2011)	27 h deprivation	Attention task	12	Left superior frontal gyrus	
Gujar et al. (2011)	32 h deprivation	Attention task	27	right posterior hippocampus, precuneus, left middle occipital gyrus	Ventral tegmental area (VTA), left putamen, amygdala, left insula
Libedinsky et al. (2011)	24 h deprivation	Attention task	22	Ventro medial PFC (vmPFC)	
Rosales-Lagarde et al. (2012)	REM/NREM sleep deprivation	Emotion reactivity task	20	NREM: right inferior, middle frontal gyri, right fusiform gyrus, left superior frontal gyrus, inferior parietal lobe, posterior middle temporal gyrus, parahippocampal gyrus REM: left anterior, posterior cingulate gyri, left posterior middle temporal gyrus	REM: left middle occipital gyrus
Lythe et al. (2012)	31 h deprivation	Working memory task	20	High-load WM: right vlPFC	Low-load WM: Right inferior parietal lobe
Dai et al. (2015)	72 h deprivation	Attention task	12	left IPL	
Wang et al. (2016)	24 h deprivation	Attention task	16	Putamen, claustrum, MFG, IPL	Cuneus
Sterpenich et al. (2017)	24 h deprivation	Verbal memory task, Verbal decision task	30	HPC, middle occipital gyrus, Precuneus	
Nir et al. (2017)	Full night sleep deprivation	Face/non-face categorization psychomotor vigilance task (cognitive lapses)	12	Medial temporal lobe (MTL) spike responses are attenuated, delayed and lengthened	
Chen et al. (2018)	24 h deprivation	Attention network task	16	Right cerebellum anterior lobe	Inferior occipital gyrus, left thalamus, insula, postcentral gyrus
Zhao et al. (2019)	24 h deprivation	Stop-signal task	22	Inferior frontal gyrus, MFG, Supplementary motor area, insula, ACC, Midcingulate cortex, Lingual gyrus, occipital cortex, fusiform gyrus, superior parietal lobule, IPL, supramarginal gyrus, Angular gyrus, Thalamus, Temporal gyrus, Subthalamic nucleus	

*WM, working memory; REM, Rapid eye movement sleep; NREM, non-rapid eye movement sleep; h, hours.*

### Parietal Lobe

Regions in the parietal lobe were also frequently linked to sleep-deprivation-induced changes in fMRI responses (Table 4). Although a few studies suggest increased neural responses under sleep-deprived conditions (Drummond et al., 2012; Lythe et al., 2012; Wang et al., 2016), most studies showed that sleep deprivation induces a reduction of neural responses in the parietal regions during cognitive tasks (Bell-McGinty et al., 2004; Habeck et al., 2004; Ninad et al., 2010; Dai et al., 2015). Specifically, sleep-loss-induced changes in the inferior parietal lobe (IPL) and the precuneus have been reliably reported. The IPL is considered to play an important role in various cognitive processes, such as spatial attention, semantic memory, and social cognition (Numssen et al., 2020). The precuneus is involved in various cognitive functions, such as episodic memory retrieval as a functional core of the default-mode network (Cavanna and Trimble, 2006; Utevsky et al., 2014). Sleep deprivation effects in these regions were observed in various cognitive processes, especially in working memory/cognitive control processes.

### Hippocampus

Neural response changes in the hippocampus have mainly been reported in long-term memory studies that utilize sleep-deprivation conditions (Gais et al., 2007; Yoo et al., 2007; Van Der Werf et al., 2009; Sterpenich et al., 2017). As noted earlier, although sleep deprivation affects memory function mainly by disrupting the memory consolidation process, behavioral changes of non-hippocampus-dependent memory indicate a negative effect of sleep deprivation on the encoding or retrieval phase.

Human fMRI studies show reductions in the neural responses of the hippocampus during memory encoding or retrieval after sleep deprivation (Gais et al., 2007; Yoo et al., 2007; Van Der Werf et al., 2009; Sterpenich et al., 2017). In addition, neural attenuation at the single neuron level was also found in the medial temporal lobe (Nir et al., 2017). One possible interpretation of the reduced neural response in the hippocampus during retrieval may be a reflection of the negative effect of sleep deprivation on memory consolidation.

### Basal Ganglia/Thalamus

While sleep deprivation is mainly associated with decreases in neural responses in the prefrontal, parietal, and hippocampal regions, increased responses under sleep-deprived conditions have mainly been reported in the basal ganglia and thalamus. This opposite direction in the neural response change in these regions can be interpreted in two ways. First, these regions are hyperactivated by extended sleep deprivation itself and irrelevant to cognitive functions. In particular, the thalamus is involved in consciousness and modulation of the sleep and wake cycle (Steriade and Llinas, 1988; Redinbaugh et al., 2020). Therefore, increased sleepiness and fatigue could require more activity to sustain consciousness. Second, this hyperactivity could be a compensatory process. Given that other regions show decreased activity after sleep deprivation, the basal ganglia and thalamus, regions which are less affected by sleep deprivation, could compensate for the lack of required neural responses.

## Influential Factors Affecting Sleep and Related Cognitive Functions

### Sleep Regulation by Homeostatic and Circadian Processes

Prior sleep studies suggest that sleep is regulated by two basic processes: a homeostatic process and a circadian process. As mentioned in the introduction, sleep deprivation can be caused by experiences such as jet lag from air travel or stressful events, which we often experience in modern society. These experiences likely affect our circadian rhythms, which can interact with the homeostatic process.

Sleep is known to show a homeostatic aspect with regard to maintaining sleep amount (Borbely and Wirz-Justice, 1982; Deboer, 2018). Thus, based on this homeostatic process, sleep deprivation or sleep restriction is usually followed by a sleep extension (Arnal et al., 2015). Moreover, EEG studies show that NREM sleep can be deepened to compensate sleep loss (Porkka-Heiskanen, 2013; Ong et al., 2016). Our sleep and wakefulness cycles also depend on endogenous circadian rhythms (Ibuka et al., 1977; Adrien et al., 1991; Åkerstedt, 2003). The suprachiasmatic nuclei (SCN) in the hypothalamus is known to play a key role in this circadian process as the primary pacemaker (Riemersma et al., 2004). This circadian process of the brain is susceptible to external timing cues called zeitgebers. The term zeitgeber (literally, time giver) refers to environmental variables, such as the light/dark cycle, the temperature, and melatonin, which are capable of acting as circadian time cues (Aschoff and Pohl, 1978; Rawashdeh and Maronde, 2012; Heyde and Oster, 2019). The circadian process regulates the timing of sleep while sleep homeostatic process influences mainly on the depth and maintenance of sleep (Deboer, 2018). Despite the fact that homeostatic and circadian processes are able to work independently, sleep models propose that the two types mutually influence each other (Borbély and Achermann, 1999; Huang et al., 2011; Fisher et al., 2013; Borbély et al., 2016). Specifically, one finding supported by strong evidence is that the circadian rhythm is less susceptible to light when the sleep homeostatic pressure is increased (Deboer, 2018). Thus, the quantity and quality of sleep can be determined by the outcome of the interaction between the circadian process and the homeostatic process, leading to different degrees of change in the associated cognitive processing.

### Influence of Light on Circadian Rhythms, Sleep, and Related Cognitive Processes

Given that disturbances in the sleep-wake cycle or sleep deprivation adversely affect cognitive functions, including long-term memory and cognitive control processes, as we discussed above (Chee and Choo, 2004; Mander et al., 2008; Ellenbogen et al., 2009; Debas et al., 2010), the modulation of circadian rhythms by controlling zeitgebers is likely to affect our cognitive functions. This modulation may contribute to the recovery of cognitive functions impaired by sleep deprivation. In this section, we discuss this possibility focusing on a powerful zeitgeber, light.

Light or the light/dark cycle is known to be the most powerful zeitgeber. It is known that the projection between retinal ganglion cells, which respond directly to light, and the SCN, which is the anterior part of the hypothalamus and which is responsible for regulating circadian rhythms and melatonin production (Berson et al., 2002; Dijk and Archer, 2009), is critical for synchronization between the circadian rhythms and the day-night cycle. In particular, a subset of mammalian (including human) retinal ganglion cells contains melanopsin, which is a type of photopigment belonging to a family of opsins (Hankins et al., 2008), and melanopsin cells play a key role in the synchronization of the circadian clocks to light, including modulation of sleep and the suppression of pineal melatonin production (Ruby et al., 2002; Tsai et al., 2009; Markwell et al., 2010; Bailes and Lucas, 2013).

Melanopsin photoreceptors are most sensitive to blue light wavelength at around 480 nm (Panda et al., 2005; Duda et al., 2020). Consistent with this, short-wavelength light exposure had the same effect on adjustments (phase shifts) of the human circadian system as white pulses that contained 185-fold more photons (Warman et al., 2003). This result suggests that the human circadian system mainly depends on the effect of short-wavelength light (Warman et al., 2003). In line with this, blue light exposure in the evening has been shown to suppress the evening onset of melatonin and lead to a phase delay of the circadian rhythm (Cajochen et al., 2005, 2011; Chellappa et al., 2011; Alkozei et al., 2016). Moreover, morning exposure to blue light is also known to suppress melatonin and leads to a phase advance of the circadian rhythm (Wright et al., 2004; Lack et al., 2007; Sletten et al., 2009).

We examined these adverse effects of sleep deprivation on cognitive functions. If controlling light exposure alters the circadian rhythm, including our sleep cycle through the connections from the retina to the SCN, subsequent changes of various cognitive functions, including memory and executive control processes, can be expected. Consistent with this expectation, exposure to higher levels of melanopic (short wavelength-enriched) white light was found to be associated with significantly less sleepiness, better working memory, better processing speeds, and better procedural learning outcomes in moderately sleep-restricted adults (Grant et al., 2021).

At present, we are often exposed to artificial light for long time, even at night, and widely used conventional LED lights have particularly strong blue light intensity (Shen et al., 2014). Therefore, disturbances in circadian rhythms and sleep deprivation are likely to be further accelerated under these conditions. Despite the beneficial effects of blue light exposure on working memory performance and alertness outcomes (Cajochen, 2007; Vandewalle et al., 2009; Chellappa et al., 2011; Esaki et al., 2016), there is still a possibility that long-term exposure to high-intensity blue light may cause disturbances in melatonin levels or the sleep cycle, which can lead to sleep deprivation and decreases in related cognitive functions. Recently, there have been efforts to improve this limitation of conventional LEDs. One example is a newly developed LED that mimics the spectrum of daylight (daylight-LED); the blue light intensity is comparable to the intensity levels of light with other wavelength ranges (Cajochen et al., 2019; Grant et al., 2021). By comparing conventional-LED and daylight-LED exposure conditions, Cajochen et al. showed that the daylight-LED has beneficial effects on sleep as well as visual comfort and alertness (Cajochen et al., 2019). Longitudinal studies in the future are needed to clarify whether these efforts based on light-source modulation can contribute to restoring disturbances in the human sleep cycle and the subsequent impairments of cognitive functions such as long-term memory processes.

## Conclusion

Prior research of human sleep deprivation generally suggests declines in long-term memory and cognitive control abilities under sleep deprivation conditions. Although the effects of sleep deprivation were tested based on different types of task paradigms and various stimuli, fairly consistent results across the studies thus far pertaining to the changes in behavioral performance outcomes and neural responses have been reported. However, there are still points that must be considered. Although the brain regions and corresponding neural response changes associated with sleep deprivation and related cognitive disruptions have been suggested, it remains still unclear as to how these neuronal changes are caused by sleep deprivation and how they disrupt cognitive processes. In particular, future studies will be needed to elucidate the direct effect of sleep deprivation on the consolidation processes of long-term memory, and to define the interaction mechanism between task difficulty and sleep deprivation considering executive control processes. Further, more in-depth research is needed to clarify the effects of environmental factors inevitably encountered in our everyday lives, such as artificial light, on our sleep and related cognitive processes.

## Author Contributions

TK, SK, MK, JK, and S-HL wrote the manuscript. TK, SK, and S-HL conceptualized and edited the manuscript. All authors contributed to the article and approved the submitted version.

## Conflict of Interest

The authors declare that the research was conducted in the absence of any commercial or financial relationships that could be construed as a potential conflict of interest.

## Publisher’s Note

All claims expressed in this article are solely those of the authors and do not necessarily represent those of their affiliated organizations, or those of the publisher, the editors and the reviewers. Any product that may be evaluated in this article, or claim that may be made by its manufacturer, is not guaranteed or endorsed by the publisher.
